# Seasonal dynamics and genetic diversity of human adenoviruses in patients with acute respiratory infection in Thailand, 2024

**DOI:** 10.1371/journal.pone.0338450

**Published:** 2025-12-09

**Authors:** Jiratchaya Puenpa, Lita Tantipraphat, Ratchadawan Aeemjinda, Preeyaporn Vichaiwattana, Sumeth Korkong, Yong Poovorawan

**Affiliations:** 1 Department of Pediatrics, Faculty of Medicine, Center of Excellence in Clinical Virology, Chulalongkorn University, Bangkok, Thailand; 2 FRS(T), The Royal Society of Thailand, Sanam Sueapa, Dusit, Bangkok, Thailand; Defense Threat Reduction Agency, UNITED STATES OF AMERICA

## Abstract

Human adenoviruses (HAdVs) are a significant cause of acute respiratory infections (ARIs), particularly in pediatric populations. Continuous molecular surveillance is essential to understand their epidemiology, genetic diversity, and seasonal dynamics. In 2024, a surveillance study was conducted in Bangkok, Thailand, involving 8,130 nasopharyngeal swabs collected from ARI patients. HAdV was detected in 5.3% (429/8,130) of ARI cases, with peak weekly positivity reaching 17.7% during weeks 10–12 (March). These HAdV-positive samples were subsequently genotyped through partial *hexon* gene sequencing and phylogenetic analysis using the maximum likelihood method. HAdV-B3 was the predominant genotype (70.7%), followed by HAdV-C2 (11.6%) and HAdV-C1 (10.4%). Genotype diversity increased toward the end of the year, with the emergence of HAdV-B7, C5, and C6. The majority of cases occurred in children aged 0–9 years, with HAdV-B3 dominating across all pediatric groups. Phylogenetic analysis revealed close genetic relationships between Thai strains and reference strains from China, Japan, the USA, and Europe, indicating both local circulation and international linkages. Despite the detection of HAdV-B7, no severe outcomes were reported in this cohort. This study also reports potential relevant sites under episodic positive selection in the *hexon* gene, suggesting adaptive evolution at specific codon positions. This study provides updated insight into the molecular epidemiology of HAdV in Thailand. The findings highlight seasonal and age-specific patterns in genotype distribution and underscore the importance of continued genomic surveillance to detect emerging variants and guide public health responses.

## Introduction

Human adenoviruses (HAdVs), classified within the *Mastadenovirus* genus of the *Adenoviridae* family, possess a linear, non-segmented, double-stranded DNA genome approximately 28–38 kilobases in length, flanked by inverted terminal repeats [1]. The *Adenoviridae* family is taxonomically divided into five genera, with *Mastadenovirus* comprising isolates from both human and nonhuman primate hosts [2]. HAdVs are non-enveloped, icosahedral viruses measuring 70–90 nm in diameter, composed of 252 capsomeres. Twelve penton capsomeres occupy the vertices and feature protruding filamentous glycoproteins with terminal knobs, while the remaining 240 *hexon* capsomeres constitute the structural faces of the capsid [2].

To date, more than 110 HAdV types have been identified and classified into seven species (A–G), initially based on serological neutralization profiles and now distinguished by their genomic features [3,4]. Recombination plays a pivotal role in HAdV evolution, particularly in cases of severe or persistent infection, and contributes to the emergence of novel genotypes with potential clinical significance [5–7].

HAdVs initiate infection by binding to specific cellular receptors on the airway or nasal epithelium. For most respiratory HAdVs, the coxsackievirus and adenovirus receptor (CAR) and/or CD46 serve as primary attachment sites, with subsequent engagement of integrins facilitating internalization via clathrin-mediated endocytosis [8,9]. Following entry, viral particles are trafficked to the nucleus where viral DNA is released, initiating early gene transcription and modulating host cell responses [10]. Infection triggers host innate immune signaling pathways, including activation of NF-κB and type I interferon responses, which coordinate antiviral defenses and influence the severity of inflammation in the respiratory epithelium [11,12]. In addition, host cell autophagy has been shown to modulate early stages of HAdV infection in airway epithelial cells, affecting viral entry, replication efficiency, and the magnitude of antiviral immune responses [13]. Variations in receptor usage, downstream signaling, and autophagic responses may collectively contribute to differences in tissue tropism, pathogenicity, and clinical outcomes among distinct HAdV types.

These molecular interactions at the cellular level underpin the broad tissue tropism exhibited by HAdVs, enabling them to infect epithelial mucosal cells across the respiratory, gastrointestinal, genitourinary, and ocular systems [14–16]. Respiratory tract infections are particularly common and can range from mild upper respiratory illnesses, such as pharyngitis and pharyngoconjunctival fever, to more severe lower respiratory conditions including bronchitis, bronchiolitis, and pneumonia, particularly among young children and immunocompromised individuals [17,18].

In Thailand, where acute respiratory infections remain a leading cause of pediatric morbidity, epidemiological surveillance of HAdVs has been limited. A study conducted between 2009 and 2012 reported a 1.0% positivity rate for HAdV among patients with respiratory tract infections, with HAdV-B3, HAdV-C1, and HAdV-C2 identified as the most frequently detected genotypes [19]. Children under the age of three accounted for the highest proportion of HAdV-positive cases, comprising 63.29% of all infections [19]. These findings highlight the pressing need for continued molecular surveillance and virological studies to clarify the burden and circulating genotypes of HAdVs in Thailand, particularly in young children.

Despite the significant public health burden posed by HAdVs, therapeutic options remain limited, with no specific antiviral agents currently approved for routine clinical use. The absence of targeted therapies underscores the urgent need for the development of effective antivirals and vaccines. Bridging this gap requires a deeper understanding of HAdV molecular biology, genetic diversity, and epidemiological dynamics to support evidence-based prevention and treatment strategies.

The specific objectives of this study were to determine the prevalence of HAdV among acute respiratory infection (ARI) cases in Thailand, identify circulating genotypes through partial hexon gene sequencing, and describe the temporal and seasonal patterns of infection. In addition, phylogenetic analysis was conducted to explore the genetic relationships between local HAdV strains and those reported globally, providing insights into molecular evolution and potential implications for public health interventions.

## Materials and methods

### Ethical statement

This study was conducted in accordance with the ethical principles outlined in the Declaration of Helsinki. Ethical approval was obtained from the Institutional Review Board of the Faculty of Medicine, Chulalongkorn University, Thailand (Approval Number: IRB0977/67). To protect patient confidentiality, all data were fully anonymized prior to analysis. Due to the retrospective nature of the study, the requirement for informed consent was waived by the Institutional Review Board. Nasopharyngeal swab specimens were accessed for research purposes on 04/03/2025. Patient demographic information, including age and sex, was obtained retrospectively from the records accompanying the nasopharyngeal swab specimens. All data were fully anonymized prior to analysis.

### Real-time PCR-based detection of HAdV in clinical samples

In 2024, a total of 8,130 nasopharyngeal swab specimens were collected from patients hospitalized with ARIs in two tertiary-care hospitals in Bangkok that have been long-term collaborators with our study team. All collected specimens were submitted to our center for comprehensive virological analysis and tested for multiple respiratory viruses, including HAdV, influenza A and B, SARS-CoV-2, respiratory syncytial virus (RSV), parainfluenza viruses, human metapneumovirus, seasonal coronaviruses, and rhinovirus, using specific real-time PCR assays for each virus. For the purpose of this study, we specifically focused on analyzing the prevalence, genotype distribution, and epidemiological characteristics of HAdV. Viral nucleic acids were extracted from 200 μL of the sample supernatant using the magLEAD 12gC automated extraction system (Precision System Science, Chiba, Japan), in accordance with the manufacturer’s instructions. This high-throughput platform ensured consistent and efficient extraction suitable for large-scale surveillance studies.

Each extracted nucleic acid sample was then subjected to real-time PCR to screen for HAdV. The assay utilized primers and probes specifically targeting the conserved region of the *hexon* gene, a key structural component of the adenoviral capsid, following previously validated protocols [20].

### HAdV genotyping

To determine the genotypes of HAdV, 429 HAdV-positive samples were randomly selected for further analysis. A conventional PCR assay was employed to amplify a portion of the *hexon* gene, producing an amplicon approximately 956 base pairs in length, using primer sequences previously published in the literature [19]. Among these 429 samples, 345 yielded amplicons of sufficient quality for successful sequencing, while the remaining samples failed to amplify or produced low-quality products, as summarized in the flow diagram (S1 Fig).

The specific primers used for amplification were ADV_F2 (5′-TTY CCC ATG GCN CAC AAC AC-3′) and ADV_R2 (5′-GYY TCR ATG AYG CCG CGG TG-3′). PCR reactions were prepared in a final volume of 25 μL, comprising 2–3 μL of extracted DNA (concentration range: 100 ng to 1 µg), 10 mM of each primer, 1X Perfect Taq MasterMix (5 PRIME, Darmstadt, Germany), and nuclease-free water. The thermocycling protocol included an initial denaturation at 94°C for 3 minutes, followed by 40 cycles of denaturation at 94°C for 30 seconds, annealing at 50°C for 30 seconds, and extension at 72°C for 1 minute and 45 seconds. A final extension step was performed at 72°C for 10 minutes. Amplicons were subsequently sent for sequencing and analysis, which were carried out by First BASE Laboratories Sdn Bhd, located in Selangor Darul Ehsan, Malaysia.

### Phylogenetic analysis

Sequence alignment was performed using the CLUSTAL W algorithm available through the European Bioinformatics Institute (EBI) web platform [21]. Phylogenetic trees were constructed using MEGA software version 10 [22], applying the maximum likelihood (ML) method. Statistical support for the tree topology was evaluated through 1,000 bootstrap replicates to ensure robustness.

To determine the most suitable nucleotide substitution model for each dataset, a model selection procedure incorporating correction factors was implemented. For the *hexon* gene analysis, the General Time Reversible (GTR) model with a proportion of invariant sites (I) and a gamma distribution (Γ) to account for rate variation among sites was selected as the best-fitting model.

### Statistical analysis

The relationship between gender and infection status, both categorical variables, was evaluated using Pearson’s chi-square test of independence. To compare infection rates across different age groups, pairwise proportion tests were applied, with the Bonferroni method used to correct for multiple comparisons. A p-value threshold of less than 0.05 was considered indicative of statistical significance. All statistical procedures were carried out using R software (version 4.4.2) [23].

### Estimation of site-specific selection pressure

To explore the evolutionary forces acting on the *hexon* gene of HAdV, we applied a comprehensive set of codon-level analytical tools that capture various modes of selection, including purifying, directional, and episodic diversifying selection. The analysis incorporated four established models: Single-Likelihood Ancestor Counting (SLAC), Fixed Effects Likelihood (FEL), Mixed Effects Model of Evolution (MEME), and the Fast Unconstrained Bayesian AppRoximation (FUBAR) [24–26]. Each method estimates site-specific ratios of non-synonymous (dN) to synonymous (dS) substitutions using either maximum likelihood (SLAC, FEL, MEME) or a Bayesian statistical framework (FUBAR), based on aligned nucleotide sequences and a corresponding phylogenetic tree. All analyses were performed via the Datamonkey web server (http://www.datamonkey.org) [27]. Positive selection was considered when identified by at least 1 method, with codons having a P threshold <0.1 or a posterior probability >0.90.

### Nucleotide sequence accession numbers

The novel sequence data generated in this study are available in the GenBank database under accession numbers PX020986–PX021330, and are publicly accessible through the NCBI GenBank database at https://www.ncbi.nlm.nih.gov/nucleotide/. Detailed accession numbers for all sequenced samples are provided in S1 Table.

## Results

### Prevalence of HAdV detection in Thailand

**Fig 1:**
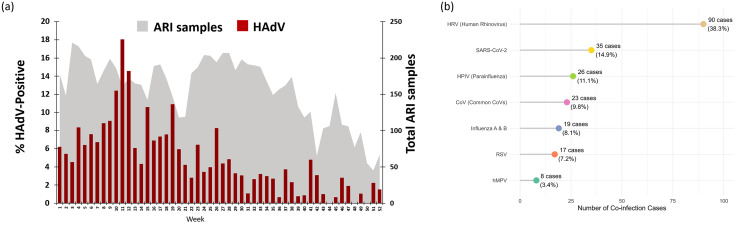
Weekly burden and proportional distribution of HAdV infections by virus group. (a) Weekly distribution of HAdV-positive samples. (b) The lollipop plot visually emphasizes both the number and proportion of cases, with color-coded markers representing virus groups.

In 2024, a surveillance study was conducted at a collaborating hospital in Bangkok to investigate the prevalence and temporal distribution of HAdV among patients presenting with ARI. A total of 8,130 nasopharyngeal swab specimens were collected and analyzed using real-time PCR. Of these, 429 samples were confirmed positive for HAdV, corresponding to an overall positivity rate of 5.3% (429/8,130), as illustrated in Fig 1a. Weekly trends in HAdV detection revealed marked temporal variability throughout the year. The weekly positivity rate ranged from 0% to a maximum of 17.7%, while the number of ARI samples tested per week varied between 45 and 214. A pronounced increase in HAdV activity was observed during the summer months, particularly between epidemiological weeks 9 and 17 (March), peaking at approximately 18% in week 12. This surge coincided with a substantial rise in ARI case numbers. Following week 41 (October to December), which corresponds to the winter season, both the number of ARI cases and HAdV-positive detections declined, although sporadic minor peaks were noted in weeks 46 and 51.

A total of 235 co-infection events were identified among HAdV-positive cases in 2024. As shown in Fig 1b, Human Rhinovirus (HRV) was the most frequently co-detected virus, accounting for 38.3% (n = 90) of co-infection cases. This was followed by SARS-CoV-2 (14.9%, n = 35), Parainfluenza viruses (HPIV; 11.1%, n = 26), and Common seasonal coronaviruses (CoVs; 9.8%, n = 23). Other co-detected viruses included Influenza A/B (8.1%, n = 19), Respiratory Syncytial Virus (RSV; 7.2%, n = 17), and Human Metapneumovirus (hMPV; 3.4%, n = 8).

**Fig 2:**
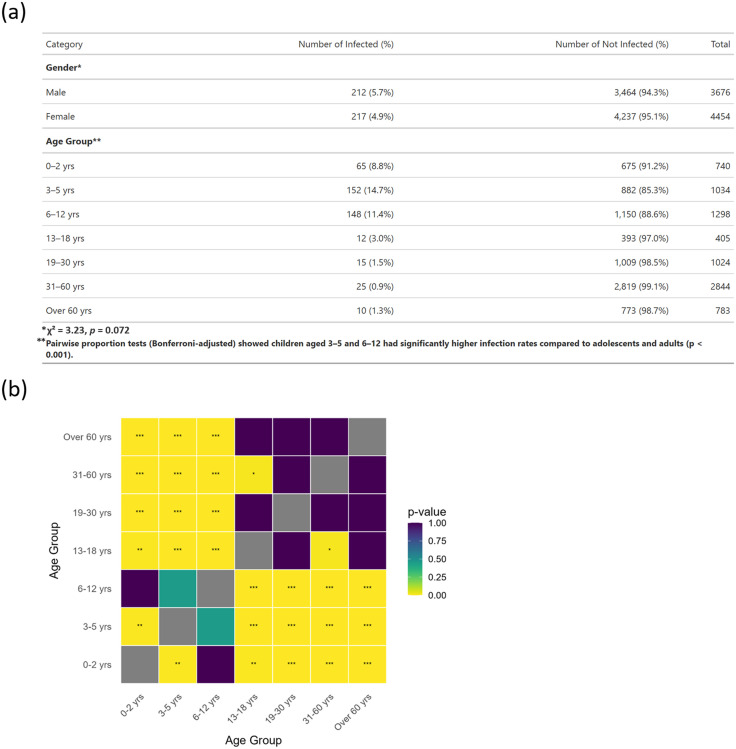
Age- and sex-specific distribution of HAdV infection and pairwise comparisons of infection rates among age groups. (a) Number and percentage of individuals with and without HAdV infection, stratified by gender and age group. “Not infected” indicates samples that tested negative for HAdV. (b) Heatmap showing pairwise comparisons of infection rates among age groups, with significant differences highlighted.

By gender, 212 of 3,676 males (5.7%) and 217 of 4,454 females (4.9%) were infected (Fig 2a). However, this difference was not statistically significant (χ² = 3.23, *p* = 0.072). In this study, age groups were classified as follows: Infants (0–2 years), Pre-school children (3–5 years), Primary school children (6–12 years), Secondary school adolescents (13–18 years), Young adults (19–30 years), Middle-aged adults (31–60 years), and Older adults (over 60 years). In contrast to gender, a significant variation in HAdV infection rates was observed across age groups (Fig 2b). Specifically, Pre-school children (14.7%) and Primary school children (11.4%) had significantly higher infection rates compared to Secondary school adolescents and all adult age groups (*p* < 0.001, Bonferroni-adjusted pairwise comparisons). No statistically significant differences in infection proportions were observed among individuals aged 13 years and older.

### Molecular characterization of the *hexon* Gene in circulating HAdV

**Fig 3:**
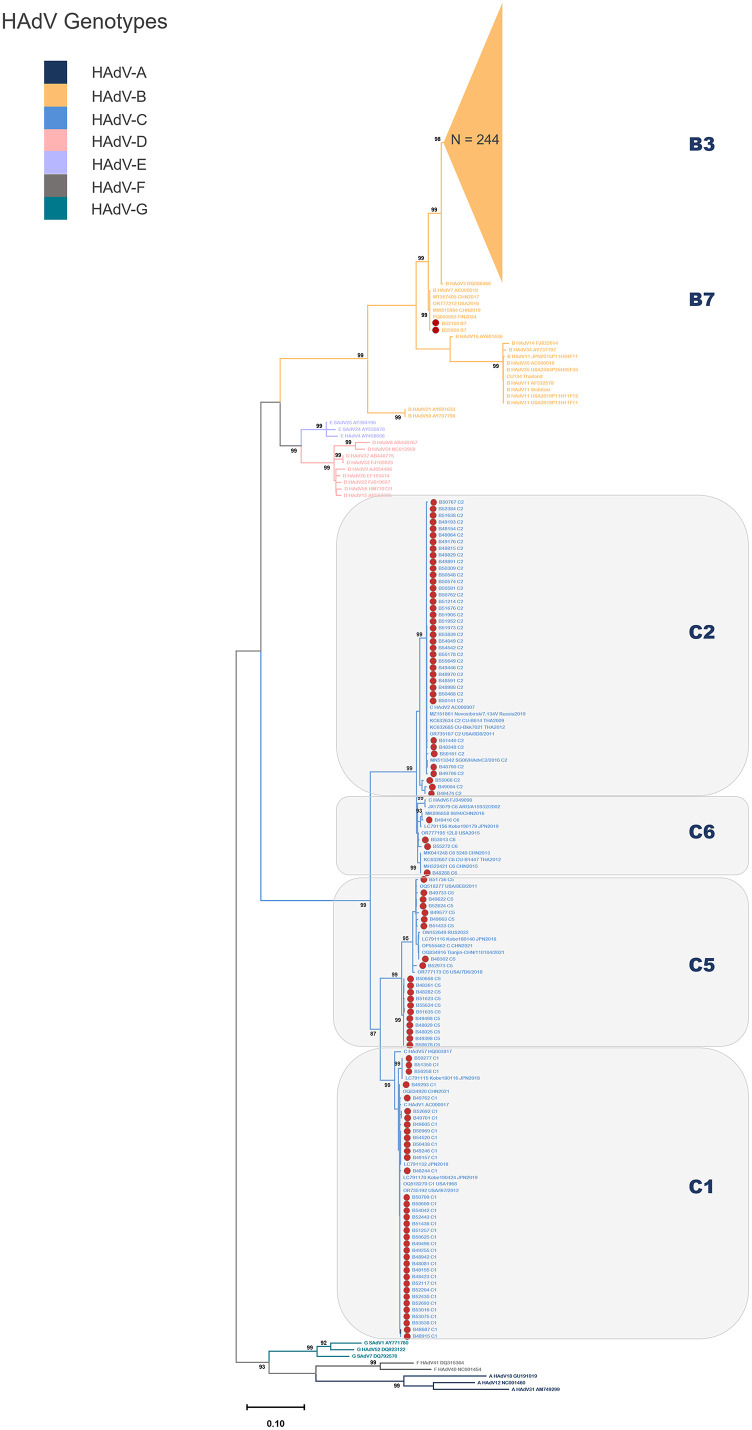
Maximum likelihood phylogenetic tree depicting the genetic relationships among Human Adenovirus (HAdV) strains identified in Thai patients with acute respiratory infections (ARIs) in 2024, based on partial *hexon* gene sequences. Representative reference strains of known HAdV genotypes are included for comparison. The scale bar indicates the number of nucleotide substitutions per site. Thai sequences are represented by red circles (n = 101), while an additional cluster of 244 Thai HAdV-B3 sequences is shown as a collapsed clade (orange triangle, N = 244). Bootstrap support values (>90%, based on 1,000 replicates) are shown at key nodes. Branch colors correspond to HAdV genotypes as indicated in the color key.

To investigate the genetic diversity of circulating human adenoviruses (HAdVs), 345 HAdV-positive clinical samples with high-quality PCR amplicons were selected for genotypic analysis. Targeting the hypervariable region of the *hexon* gene, partial sequences were amplified using conventional PCR. Phylogenetic analysis was performed to assess genetic relationships between the circulating strains and reference sequences obtained from the GenBank database. Among the successfully genotyped samples (n = 345; Fig 3), HAdV-B3 was the predominant genotype, representing 70.7% of cases. Additional genotypes included HAdV-C2 (11.3%), HAdV-C1 (10.4%), HAdV-C5 (5.8%), HAdV-C6 (1.2%), and HAdV-B7 (0.6%).

Phylogenetic analysis revealed that the HAdV-B3 strains formed a closely related cluster with reference strains previously reported from China (2015), the USA (2011), Japan (2023), and Germany (2023), suggesting global dissemination and genetic conservation. The two HAdV-B7 strains identified in this study (B52150 and B55900) were closely related to strains reported from Finland in 2024. Most HAdV-C1 Thai isolates clustered with strains from the USA (2012), China (2021), and Japan (2018–2019). Notably, a distinct subcluster composed of three Thai strains (B50277, B51350, and B50258) was observed alongside a genetically similar strain from Japan (2018), indicating possible regional lineage divergence.

HAdV-C5 strains segregated into two distinct phylogenetic groups: one composed exclusively of 11 Thai strains from this study, and another including nine Thai strains clustered with reference sequences from the USA, Russia, China, and Japan. Most HAdV-C2 strains grouped with reference strains from Singapore (2016), Russia (2019), and the USA (2011); however, three isolates (B55066, B49004, and B49474) formed a separate lineage. All four Thai HAdV-C6 strains clustered tightly and showed close phylogenetic relationships with strains from Argentina (2002), Japan (2019), the USA (2015), and China (2015), reflecting a potentially conserved but globally dispersed lineage.

### Genotypic trends by month and age

**Fig 4:**
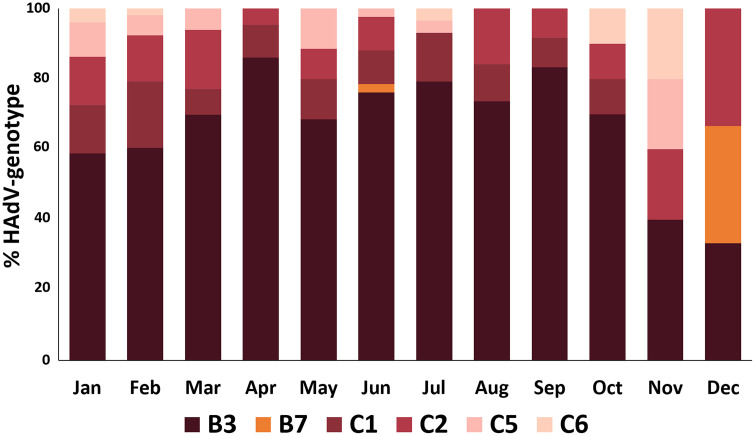
Analysis of HAdVs Genotypes Identified in Bangkok, 2024.

The monthly distribution of HAdV genotypes throughout 2024 is illustrated in Fig 4. HAdV-B3 remained the most consistently detected genotype across the year, comprising 60–80% of circulating strains from January through September. Other genotypes, including C1, C2, C5, and C6, were detected at lower frequencies and showed intermittent circulation. A notable shift in genotype composition emerged in the final quarter of the year. In October, B3 declined in relative prevalence, coinciding with an increase in C5 and C6. This trend intensified in November and December, where genotype diversity increased markedly. During these months, B3 accounted for less than 50% of cases, while B7 reemerged prominently in November, and C5 and C6 demonstrated elevated proportions through the end of the year.

**Fig 5:**
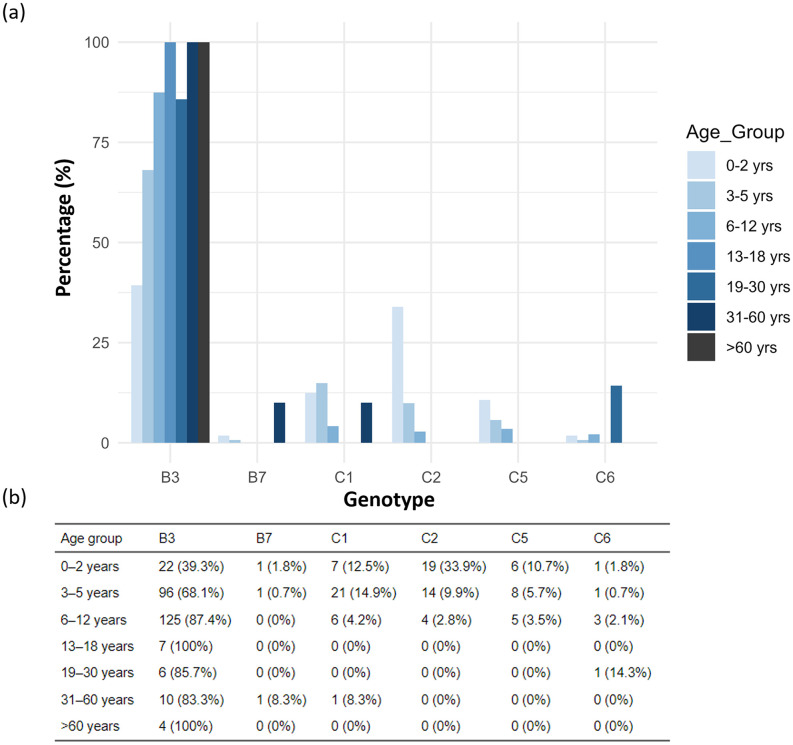
Age-specific distribution of human adenovirus (HAdV) genotypes. (a) Distribution of human adenovirus (HAdV) genotypes by age group. (b) The number and percentage of HAdV genotypes stratified by age group.

Among the 345 HAdV-positive cases, genotype B3 was the most prevalent across all age groups, particularly in children (Fig 5a and 5b). It accounted for the majority of infections in Primary school children (87.4%), Pre-school children (68.1%), and Infants (39.3%). Notably, B3 was the sole genotype detected in Secondary school adolescents (100%) and Older adults (100%), and was dominant in Young adults (85.7%) and Middle-aged adults (83.3%).

Other genotypes, including C1, C2, C5, and C6, were detected at lower frequencies and primarily among younger age groups. For instance, genotype C2 was found in 33.9% of Infants and 9.9% of Pre-school children, while genotype C1 was most common in Pre-school children (14.9%) and Infants (12.5%). Genotypes C5 and C6 were rarely detected and only in children, with proportions below 11%. Genotype B7 was infrequently detected overall, identified in only three cases: one each in Infants, Pre-school children, and Middle-aged adults. These findings indicate a clear age-related distribution of HAdV genotypes, with greater genotype diversity observed among Infants and Pre-school children, and B3 predominating in older age groups.

### Site-specific selective pressure acting on the *hexon* gene

To investigate the adaptive molecular evolution of HAdV, codon-specific selective pressures acting on the *hexon* gene were examined by estimating the ratio of non-synonymous (dN) to synonymous (dS) substitution rates across the phylogeny. A suite of complementary codon-based models implemented in multiple selection detection algorithms (SLAC, FEL, MEME, and FUBAR) was employed.

**Fig 6:**
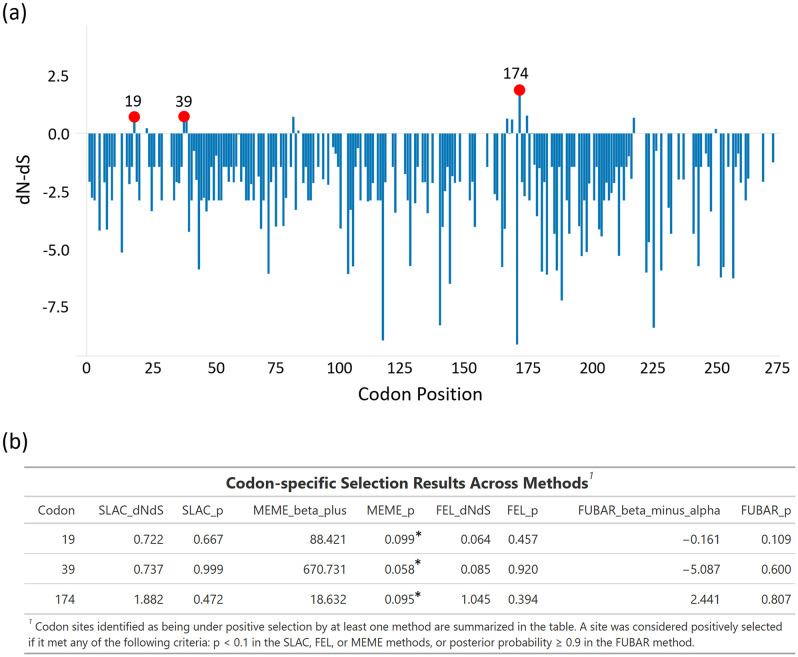
Site-specific selection pressure on the hexon gene of human adenoviruses (HAdVs). (a) Overview of site-specific selection pressure across the partial hexon region of human adenoviruses (HAdVs), as estimated by SLAC analysis. Bars represent the evolutionary rate (dN−dS) for each codon. Red circles highlight codon positions identified as statistically significant for selection. Codons were classified as under positive or negative selection based on evidence from at least one analytical method. (b) Selection pressure analysis of the hexon protein (277 codons) of HAdV using SLAC, FEL, IFEL, MEME, and FUBAR methods (www.datamonkey.org). Asterisks (*) denote statistically significant sites.

Using SLAC (Fig 6a), the overall dN/dS ratio across the *hexon* region was estimated at 0.095, with 45 codons showing significantly lower dN than dS (dN/dS < 1, p ≤ 0.1). FEL identified 157 codons with significant evidence of purifying selection (p ≤ 0.1), while FUBAR confirmed pervasive purifying selection at 153 codons (posterior probability ≥ 0.9), with no evidence of pervasive positive selection.

In contrast, MEME detected three codon positions (19, 39, and 174) that were evolving under episodic diversifying selection (p ≤ 0.1) (Fig 6b).

## Discussion

To better understand the epidemiology of human adenovirus (HAdV) infections, this study analyzed the genetic profiles of HAdV strains from ARI patients in Bangkok in 2024. We assessed seasonal patterns, genotype distribution, and phylogenetic relationships to clarify local transmission dynamics. Weekly surveillance data and genotype analysis revealed insights into strain prevalence and evolution. Comparison with global reference sequences highlighted the genetic links between Thai and international HAdV strains, suggesting both local circulation and global connectivity of emerging variants.

The detection of HAdV in 5.3% of ARI cases in this study underscores its continued relevance as a respiratory pathogen in the Thai population. Although this prevalence is lower than many reports from other regions, it still reflects a measurable burden. For instance, studies in India have documented positivity rates ranging from 6.8% to as high as 18.6% [28,29], while findings from China report slightly higher rates than ours, between 6.9% and 10.8% [30–32]. Significantly elevated rates are often associated with closed or outbreak-prone settings; for example, the Korean military reported a 36.0% positivity rate during outbreaks [33], and in northern Vietnam, a large pediatric outbreak at the end of 2022 showed HAdV in 54.5% of cases [34]. Additionally, surveillance data from the United States National Adenovirus Type Reporting System (NATRS) indicated a positivity rate of up to 77.0% among submitted specimens from 2017 to 2023, reflecting continued virus circulation despite reduced testing volume post-COVID-19 [35]. Such disparities may result from differences in surveillance strategies, testing policies, seasonal trends, and population characteristics across regions.

The HAdV outbreak in Bangkok peaked between epidemiological weeks 10 and 12 of 2024, with the highest detection rate of 20.3% reported in March. This seasonal pattern aligns with regional variations observed globally. In southern China, peak HAdV activity was documented earlier, in January 2024, while in Jining City, China, the peak occurred later, in July 2024 [32,36]. Similarly, in the Republic of Korea, two distinct peaks were observed, occurring in August–September 2023 and April–May 2024, respectively, suggesting multiple waves of transmission [37]. In the United Kingdom, HAdV cases peaked in April 2024 [38], while in Wales, the peak was reported in January of the same year, highlighting intra-country variability. In contrast, Australia reported sporadic HAdV detections throughout the year, without a clear seasonal peak [39]. These temporal variations likely reflect climatic and population factors, suggesting a seasonal pattern of HAdV circulation in Bangkok and emphasizing the need for continued surveillance.

Co-infections with multiple respiratory viruses were detected in over half of the HAdV-positive cases in this study. Such mixed infections are frequently reported in acute respiratory illness and may influence viral interactions, transmissibility, and disease outcomes such as pneumonia, intensive care unit (ICU) admission, prolonged hospital stay, and death [40]. Although clinical data were not collected to evaluate the clinical severity associated with co-infection, the high frequency observed here suggests that HAdV often circulates concurrently with other respiratory pathogens. This finding highlights the complexity of attributing acute respiratory symptoms to a single viral agent and emphasizes the need for integrated molecular and clinical surveillance in future studies.

In this study, the highest proportion of HAdV cases was observed in the 5–9 years age group, followed by children aged 0–4 years. This age distribution aligns with findings from previous studies that highlight the vulnerability of young children to HAdV infections. For example, a recent study from China (2023–2024) reported that 59.5% of cases occurred in children aged 0–6 years, with species B adenoviruses showing a higher infection rate among school-aged children [41]. Additionally, a severe outbreak in a nursery in Dakar in April 2024 involved four infants and was associated with subgroup B1 strains, underscoring the potential for significant HAdV transmission and disease severity in younger age groups [42].

The predominant genotype identified in this study was HAdV-B3, followed by HAdV-C2 and HAdV-C1. HAdV-B3 is well-documented as a major cause of respiratory illness in children and has been frequently implicated in outbreaks across various regions [34]. In contrast, HAdV-B7, though less prevalent, is often associated with more severe clinical outcomes. Interestingly, our data also indicate a seasonal pattern in genotype distribution, with greater heterogeneity and the emergence of less common genotypes during the colder months. Notably, a 2013–2014 outbreak in the United States reported HAdV-B7 infections with intensive care unit (ICU) admission rates reaching 46% [43], and a 2024 outbreak in Finland caused unusually severe respiratory illness among military conscripts, resulting in six deaths [44]. While many prior studies have reported severe illness associated with HAdV-B7 [43,44], particularly in very young children or military recruits, community-wide outbreaks involving a broader pediatric population have been less frequently documented. In this study, only two HAdV-B7 strains were identified among more than three hundred genotyped specimens, and most participants were aged 3–12 years. The absence of reported severe cases may therefore reflect both the limited number of B7 detections and the study’s design, in which detailed clinical data were not systematically collected. Additionally, strain-specific genetic variations could influence viral virulence and clinical outcomes. Future investigations combining comprehensive clinical information with full-genome characterization of circulating B7 strains are warranted to elucidate these relationships.

Comparison with recent systematic reviews highlights regional heterogeneity in adenovirus circulation. A meta-analysis from China reported winter peaks in northern regions, dual peaks in southern regions, and HAdV-7/B55 as predominant types among adults [45]. In contrast, our Bangkok data show lower overall positivity, predominance of HAdV-B3, and higher infection rates among preschool and primary school children. These differences likely reflect climatic variation, host immunity, and population characteristics, emphasizing the need for region-specific surveillance.

In the current study, a predominant signal of purifying selection was observed across the *hexon* region, as indicated by the low overall dN/dS ratio (0.095) estimated by SLAC and the large number of codons under significant purifying selection identified by FEL (157 codons) and confirmed by FUBAR (153 codons). This widespread negative selection likely reflects the functional constraints on the *hexon* protein, which is essential for viral structure and immune recognition. Notably, despite the dominance of purifying selection, MEME detected episodic diversifying selection at three codon positions (19, 39, and 174). Codons 19 and 39 in our amplified sequences correspond to positions 650 and 670 in the reference hexon protein, located within the viral jellyroll connector (VC) domain, which is important for *hexon* trimer assembly and capsid stability [46]. Codon 174 corresponds to position 805 in the FG2 loop, a surface-exposed region contributing to antigenicity [46]. Variation at these sites may therefore influence capsid stability or immune recognition, suggesting potential adaptive significance. These findings partially contrast with those of Saha et al., who reported no evidence of positive selection in either the *hexon* or fiber genes, though purifying selection was also observed [28]. Conversely, a study from Taiwan on HAdV-3 and HAdV-7 reported positive selection sites within the *hexon* protein, consistent with our findings [47]. Such intertype and regional variations, together with the identified positively selected sites, highlight the complex evolutionary dynamics and potential adaptive changes within the adenovirus *hexon* gene, warranting further functional investigation.

This study has several limitations that should be considered. First, only a partial region of the *hexon* gene was amplified using previously published primers, which may not equally detect all genetic variants. This limitation might have influenced the detection of certain HAdV strains, the observed genotype distribution, and the identification of recombinant forms. Comprehensive genomic characterization, including sequencing of the penton base and fiber genes, would be required to more accurately assess recombination events and viral evolution. Second, clinical data were not collected alongside the molecular findings, which restricts our ability to correlate specific genotypes with clinical severity, such as pneumonia or other severe outcomes. Third, this study focused exclusively on HAdV detected in acute respiratory illness. Including samples from other clinical syndromes, such as gastroenteritis or conjunctivitis, would provide a more complete picture of genotype distribution across different disease presentations and help understand the broader impact of HAdV in the population. Fourth, the study was conducted over a single one-year period, which may limit the generalizability of the findings to other years or seasonal variations.

In conclusion, the present study enhances our understanding of the genomic epidemiology and evolution of human adenoviruses (HAdVs) in Bangkok, Thailand. The dominant circulating genotype identified was HAdV-B3, with a marked increase in activity beginning in March 2024. Phylogenetic analysis revealed multiple distinct clusters within each genotype, suggesting several independent introductions and ongoing local transmission. Our findings demonstrate the value of molecular epidemiology in tracking HAdV diversity and identifying emerging genotypes, which may inform public health strategies and help mitigate the impact of future outbreaks.

## Supporting information

S1 Fig Flow diagram summarizing sample selection and sequencing outcomes for human adenovirus (HAdV) genotyping. (PDF)

S1 Table NCBI accession numbers for the *hexon* gene of human adenovirus collected in this study. (PDF)
