# Supplementary material for: Seasonal dynamics and genetic diversity of human adenoviruses in patients with acute respiratory infection in Thailand, 2024
Source: PLoS One. 2025 Dec 9;20(12):e0338450. doi: 10.1371/journal.pone.0338450 (PMC12688151; doi:10.1371/journal.pone.0338450)

Supplement Figure 1. Flow diagram summarizing sample selection and sequencing outcomes for human adenovirus (HAdV) genotyping.

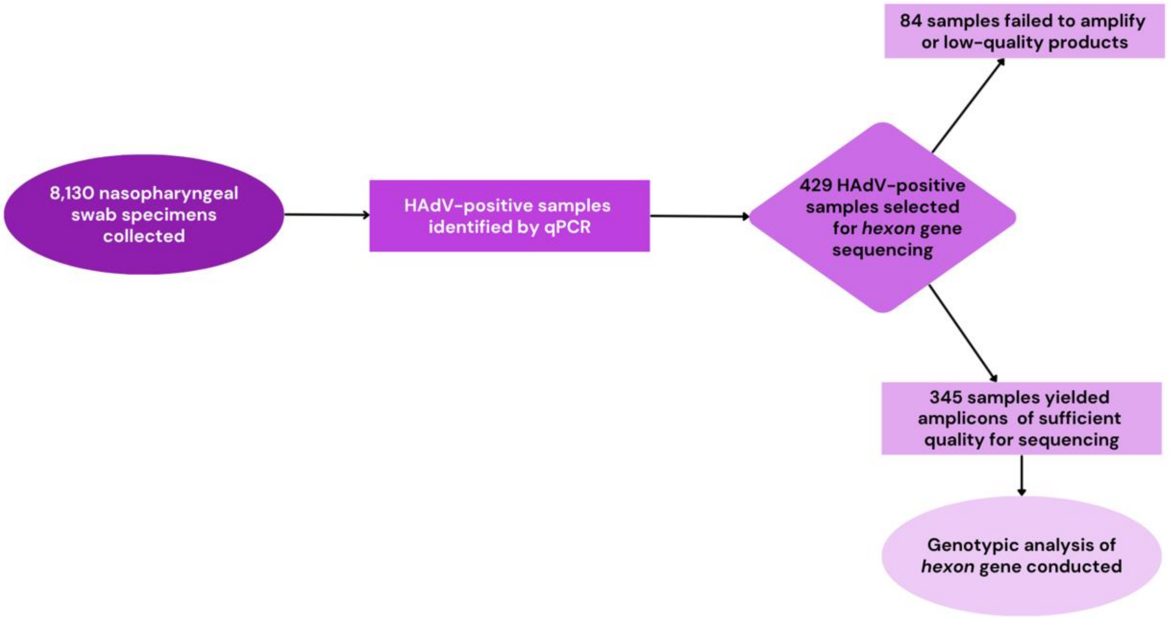

Supplement: S1 Fig — (PDF) [file pone.0338450.s001.pdf]
