# Supplementary material for: Seasonal dynamics and genetic diversity of human adenoviruses in patients with acute respiratory infection in Thailand, 2024
Source: PLoS One. 2025 Dec 9;20(12):e0338450. doi: 10.1371/journal.pone.0338450 (PMC12688151; doi:10.1371/journal.pone.0338450)
Supplement: S1 Table — (PDF) [file pone.0338450.s002.pdf]

Supplement Table 1. NCBI Accession Numbers for the *hexon* gene of human adenovirus collected in this study.

No.	Code	Specimen type	Host	Country	Collection date	Accesion number
Seq1	B48288	Nasal swab	Homo sapiens	Thailand	10-Jan-24	PX020986
Seq2	B55272	Nasal swab	Homo sapiens	Thailand	24-Oct-24	PX020987
Seq3	B53013	Nasal swab	Homo sapiens	Thailand	13-Jul-24	PX020988
Seq4	B50161	Nasal swab	Homo sapiens	Thailand	25-Apr-24	PX020989
Seq5	B49706	Nasal swab	Homo sapiens	Thailand	10-Mar-24	PX020990
Seq6	B49416	Nasal swab	Homo sapiens	Thailand	21-Feb-24	PX020991
Seq7	B49004	Nasal swab	Homo sapiens	Thailand	05-Feb-24	PX020992
Seq8	B55066	Nasal swab	Homo sapiens	Thailand	07-Oct-24	PX020993
Seq9	B48700	Nasal swab	Homo sapiens	Thailand	25-Jan-24	PX020994
Seq10	B49474	Nasal swab	Homo sapiens	Thailand	23-Feb-24	PX020995
Seq11	B50767	Nasal swab	Homo sapiens	Thailand	27-Mar-24	PX020996
Seq12	B50141	Nasal swab	Homo sapiens	Thailand	23-Apr-24	PX020997
Seq13	B50468	Nasal swab	Homo sapiens	Thailand	05-Mar-24	PX020998
Seq14	B48348	Nasal swab	Homo sapiens	Thailand	14-Jan-24	PX020999
Seq15	B51440	Nasal swab	Homo sapiens	Thailand	13-May-24	PX021000
Seq16	B48988	Nasal swab	Homo sapiens	Thailand	04-Feb-24	PX021001
Seq17	B48591	Nasal swab	Homo sapiens	Thailand	22-Jan-24	PX021002
Seq18	B48970	Nasal swab	Homo sapiens	Thailand	04-Feb-24	PX021003
Seq19	B49446	Nasal swab	Homo sapiens	Thailand	22-Feb-24	PX021004
Seq20	B55649	Nasal swab	Homo sapiens	Thailand	12-Nov-24	PX021005
Seq21	B55178	Nasal swab	Homo sapiens	Thailand	14-Oct-24	PX021006
Seq22	B54542	Nasal swab	Homo sapiens	Thailand	12-Sep-24	PX021007
Seq23	B54049	Nasal swab	Homo sapiens	Thailand	20-Aug-24	PX021008
Seq24	B53839	Nasal swab	Homo sapiens	Thailand	13-Aug-24	PX021009
Seq25	B51973	Nasal swab	Homo sapiens	Thailand	08-Jun-24	PX021010
Seq26	B51952	Nasal swab	Homo sapiens	Thailand	07-Jun-24	PX021011
Seq27	B51905	Nasal swab	Homo sapiens	Thailand	05-Jun-24	PX021012
Seq28	B51676	Nasal swab	Homo sapiens	Thailand	27-May-24	PX021013
Seq29	B51214	Nasal swab	Homo sapiens	Thailand	27-Apr-24	PX021014
Seq30	B50762	Nasal swab	Homo sapiens	Thailand	27-Mar-24	PX021015
Seq31	B50581	Nasal swab	Homo sapiens	Thailand	14-Mar-24	PX021016
Seq32	B50574	Nasal swab	Homo sapiens	Thailand	14-Mar-24	PX021017
Seq33	B50548	Nasal swab	Homo sapiens	Thailand	12-Mar-24	PX021018
Seq34	B50309	Nasal swab	Homo sapiens	Thailand	10-May-24	PX021019
Seq35	B49891	Nasal swab	Homo sapiens	Thailand	29-Mar-24	PX021020
Seq36	B49829	Nasal swab	Homo sapiens	Thailand	23-Mar-24	PX021021
Seq37	B49815	Nasal swab	Homo sapiens	Thailand	21-Mar-24	PX021022

Seq38	B49176	Nasal swab	Homo sapiens	Thailand	11-Feb-24	PX021023
Seq39	B48064	Nasal swab	Homo sapiens	Thailand	03-Jan-24	PX021024
Seq40	B48154	Nasal swab	Homo sapiens	Thailand	06-Jan-24	PX021025
Seq41	B49193	Nasal swab	Homo sapiens	Thailand	11-Feb-24	PX021026
Seq42	B51638	Nasal swab	Homo sapiens	Thailand	25-May-24	PX021027
Seq43	B52384	Nasal swab	Homo sapiens	Thailand	19-Jun-24	PX021028
Seq44	B52973	Nasal swab	Homo sapiens	Thailand	12-Jul-24	PX021029
Seq45	B48302	Nasal swab	Homo sapiens	Thailand	12-Jan-24	PX021030
Seq46	B51433	Nasal swab	Homo sapiens	Thailand	13-May-24	PX021031
Seq47	B51736	Nasal swab	Homo sapiens	Thailand	29-May-24	PX021032
Seq48	B49733	Nasal swab	Homo sapiens	Thailand	13-Mar-24	PX021033
Seq49	B49577	Nasal swab	Homo sapiens	Thailand	27-Feb-24	PX021034
Seq50	B48282	Nasal swab	Homo sapiens	Thailand	10-Jan-24	PX021035
Seq51	B50656	Nasal swab	Homo sapiens	Thailand	12-Mar-24	PX021036
Seq52	B48361	Nasal swab	Homo sapiens	Thailand	15-Jan-24	PX021037
Seq53	B49498	Nasal swab	Homo sapiens	Thailand	24-Feb-24	PX021038
Seq54	B48029	Nasal swab	Homo sapiens	Thailand	01-Jan-24	PX021039
Seq55	B48025	Nasal swab	Homo sapiens	Thailand	01-Jan-24	PX021040
Seq56	B49398	Nasal swab	Homo sapiens	Thailand	20-Feb-24	PX021041
Seq57	B50678	Nasal swab	Homo sapiens	Thailand	20-Mar-24	PX021042
Seq58	B51635	Nasal swab	Homo sapiens	Thailand	25-May-24	PX021043
Seq59	B51623	Nasal swab	Homo sapiens	Thailand	24-May-24	PX021044
Seq60	B49663	Nasal swab	Homo sapiens	Thailand	06-Mar-24	PX021045
Seq61	B49622	Nasal swab	Homo sapiens	Thailand	02-Mar-24	PX021046
Seq62	B52624	Nasal swab	Homo sapiens	Thailand	30-Jun-24	PX021047
Seq63	B55624	Nasal swab	Homo sapiens	Thailand	15-Nov-24	PX021048
Seq64	B50258	Nasal swab	Homo sapiens	Thailand	05-May-24	PX021049
Seq65	B50277	Nasal swab	Homo sapiens	Thailand	07-May-24	PX021050
Seq66	B51350	Nasal swab	Homo sapiens	Thailand	07-May-24	PX021051
Seq67	B48687	Nasal swab	Homo sapiens	Thailand	24-Jan-24	PX021052
Seq68	B48915	Nasal swab	Homo sapiens	Thailand	01-Feb-24	PX021053
Seq69	B52692	Nasal swab	Homo sapiens	Thailand	02-Jul-24	PX021054
Seq70	B50709	Nasal swab	Homo sapiens	Thailand	23-Mar-24	PX021055
Seq71	B50609	Nasal swab	Homo sapiens	Thailand	17-Mar-24	PX021056
Seq72	B48244	Nasal swab	Homo sapiens	Thailand	09-Jan-24	PX021057
Seq73	B49701	Nasal swab	Homo sapiens	Thailand	10-Mar-24	PX021058
Seq74	B49762	Nasal swab	Homo sapiens	Thailand	16-Mar-24	PX021059
Seq75	B49605	Nasal swab	Homo sapiens	Thailand	29-Feb-24	PX021060
Seq76	B49157	Nasal swab	Homo sapiens	Thailand	10-Feb-24	PX021061
Seq77	B49246	Nasal swab	Homo sapiens	Thailand	14-Feb-24	PX021062
Seq78	B50438	Nasal swab	Homo sapiens	Thailand	04-Mar-24	PX021063

Seq79	B50969	Nasal swab	Homo sapiens	Thailand	13-Apr-24	PX021064
Seq80	B54520	Nasal swab	Homo sapiens	Thailand	10-Sep-24	PX021065
Seq81	B54042	Nasal swab	Homo sapiens	Thailand	21-Aug-24	PX021066
Seq82	B52443	Nasal swab	Homo sapiens	Thailand	24-Jun-24	PX021067
Seq83	B51436	Nasal swab	Homo sapiens	Thailand	13-May-24	PX021068
Seq84	B51257	Nasal swab	Homo sapiens	Thailand	29-Apr-24	PX021069
Seq85	B50625	Nasal swab	Homo sapiens	Thailand	18-Mar-24	PX021070
Seq86	B49496	Nasal swab	Homo sapiens	Thailand	24-Feb-24	PX021071
Seq87	B49293	Nasal swab	Homo sapiens	Thailand	15-Feb-24	PX021072
Seq88	B49255	Nasal swab	Homo sapiens	Thailand	14-Feb-24	PX021073
Seq89	B48942	Nasal swab	Homo sapiens	Thailand	02-Feb-24	PX021074
Seq90	B48081	Nasal swab	Homo sapiens	Thailand	03-Jan-24	PX021075
Seq91	B48155	Nasal swab	Homo sapiens	Thailand	06-Jan-24	PX021076
Seq92	B48423	Nasal swab	Homo sapiens	Thailand	17-Jan-24	PX021077
Seq93	B52117	Nasal swab	Homo sapiens	Thailand	12-Jun-24	PX021078
Seq94	B52204	Nasal swab	Homo sapiens	Thailand	15-Jun-24	PX021079
Seq95	B52430	Nasal swab	Homo sapiens	Thailand	23-Jun-24	PX021080
Seq96	B52693	Nasal swab	Homo sapiens	Thailand	02-Jul-24	PX021081
Seq97	B53016	Nasal swab	Homo sapiens	Thailand	14-Jul-24	PX021082
Seq98	B53075	Nasal swab	Homo sapiens	Thailand	15-Jul-24	PX021083
Seq99	B53538	Nasal swab	Homo sapiens	Thailand	01-Aug-24	PX021084
Seq100	B56092	Nasal swab	Homo sapiens	Thailand	26-Dec-24	PX021085
Seq101	B54238	Nasal swab	Homo sapiens	Thailand	29-Aug-24	PX021086
Seq102	B54162	Nasal swab	Homo sapiens	Thailand	25-Aug-24	PX021087
Seq103	B52258	Nasal swab	Homo sapiens	Thailand	17-Jun-24	PX021088
Seq104	B51900	Nasal swab	Homo sapiens	Thailand	05-Jun-24	PX021089
Seq105	B51546	Nasal swab	Homo sapiens	Thailand	20-May-24	PX021090
Seq106	B51024	Nasal swab	Homo sapiens	Thailand	16-Apr-24	PX021091
Seq107	B49523	Nasal swab	Homo sapiens	Thailand	25-Feb-24	PX021092
Seq108	B49516	Nasal swab	Homo sapiens	Thailand	25-Feb-24	PX021093
Seq109	B49149	Nasal swab	Homo sapiens	Thailand	10-Feb-24	PX021094
Seq110	B48143	Nasal swab	Homo sapiens	Thailand	05-Jan-24	PX021095
Seq111	B48973	Nasal swab	Homo sapiens	Thailand	04-Feb-24	PX021096
Seq112	B49038	Nasal swab	Homo sapiens	Thailand	06-Feb-24	PX021097
Seq113	B55035	Nasal swab	Homo sapiens	Thailand	06-Oct-24	PX021098
Seq114	B51324	Nasal swab	Homo sapiens	Thailand	04-May-24	PX021099
Seq115	B51092	Nasal swab	Homo sapiens	Thailand	20-Apr-24	PX021100
Seq116	B49032	Nasal swab	Homo sapiens	Thailand	06-Feb-24	PX021101
Seq117	B48625	Nasal swab	Homo sapiens	Thailand	23-Jan-24	PX021102
Seq118	B48653	Nasal swab	Homo sapiens	Thailand	24-Jan-24	PX021103
Seq119	B49501	Nasal swab	Homo sapiens	Thailand	24-Feb-24	PX021104

Seq120	B51142	Nasal swab	Homo sapiens	Thailand	23-Apr-24	PX021105
Seq121	B51243	Nasal swab	Homo sapiens	Thailand	28-Apr-24	PX021106
Seq122	B52233	Nasal swab	Homo sapiens	Thailand	16-Jun-24	PX021107
Seq123	B52577	Nasal swab	Homo sapiens	Thailand	28-Jun-24	PX021108
Seq124	B52316	Nasal swab	Homo sapiens	Thailand	21-Jun-24	PX021109
Seq125	B52279	Nasal swab	Homo sapiens	Thailand	18-Jun-24	PX021110
Seq126	B51311	Nasal swab	Homo sapiens	Thailand	03-May-24	PX021111
Seq127	B51083	Nasal swab	Homo sapiens	Thailand	19-Apr-24	PX021112
Seq128	B48655	Nasal swab	Homo sapiens	Thailand	24-Jan-24	PX021113
Seq129	B48223	Nasal swab	Homo sapiens	Thailand	08-Jan-24	PX021114
Seq130	B48308	Nasal swab	Homo sapiens	Thailand	12-Jan-24	PX021115
Seq131	B48514	Nasal swab	Homo sapiens	Thailand	19-Jan-24	PX021116
Seq132	B48660	Nasal swab	Homo sapiens	Thailand	24-Jan-24	PX021117
Seq133	B49330	Nasal swab	Homo sapiens	Thailand	17-Feb-24	PX021118
Seq134	B51046	Nasal swab	Homo sapiens	Thailand	18-Apr-24	PX021119
Seq135	B49396	Nasal swab	Homo sapiens	Thailand	20-Feb-24	PX021120
Seq136	B49772	Nasal swab	Homo sapiens	Thailand	17-Mar-24	PX021121
Seq137	B49862	Nasal swab	Homo sapiens	Thailand	26-Mar-24	PX021122
Seq138	B49915	Nasal swab	Homo sapiens	Thailand	31-Mar-24	PX021123
Seq139	B50544	Nasal swab	Homo sapiens	Thailand	11-Mar-24	PX021124
Seq140	B50556	Nasal swab	Homo sapiens	Thailand	13-Mar-24	PX021125
Seq141	B50586	Nasal swab	Homo sapiens	Thailand	14-Mar-24	PX021126
Seq142	B50676	Nasal swab	Homo sapiens	Thailand	20-Mar-24	PX021127
Seq143	B50713	Nasal swab	Homo sapiens	Thailand	23-Mar-24	PX021128
Seq144	B49911	Nasal swab	Homo sapiens	Thailand	31-Mar-24	PX021129
Seq145	B49931	Nasal swab	Homo sapiens	Thailand	02-Apr-24	PX021130
Seq146	B50328	Nasal swab	Homo sapiens	Thailand	27-Feb-24	PX021131
Seq147	B49727	Nasal swab	Homo sapiens	Thailand	13-Mar-24	PX021132
Seq148	B50018	Nasal swab	Homo sapiens	Thailand	11-Apr-24	PX021133
Seq149	B50312	Nasal swab	Homo sapiens	Thailand	10-May-24	PX021134
Seq150	B50445	Nasal swab	Homo sapiens	Thailand	04-Mar-24	PX021135
Seq151	B50534	Nasal swab	Homo sapiens	Thailand	11-Mar-24	PX021136
Seq152	B50562	Nasal swab	Homo sapiens	Thailand	13-Mar-24	PX021137
Seq153	B50672	Nasal swab	Homo sapiens	Thailand	20-Mar-24	PX021138
Seq154	B50722	Nasal swab	Homo sapiens	Thailand	24-Mar-24	PX021139
Seq155	B50572	Nasal swab	Homo sapiens	Thailand	14-Mar-24	PX021140
Seq156	B50002	Nasal swab	Homo sapiens	Thailand	09-Apr-24	PX021141
Seq157	B49995	Nasal swab	Homo sapiens	Thailand	08-Apr-24	PX021142
Seq158	B49401	Nasal swab	Homo sapiens	Thailand	20-Feb-24	PX021143
Seq159	B49403	Nasal swab	Homo sapiens	Thailand	20-Feb-24	PX021144
Seq160	B49752	Nasal swab	Homo sapiens	Thailand	15-Mar-24	PX021145

Seq161	B49753	Nasal swab	Homo sapiens	Thailand	15-Mar-24	PX021146
Seq162	B49853	Nasal swab	Homo sapiens	Thailand	25-Mar-24	PX021147
Seq163	B50344	Nasal swab	Homo sapiens	Thailand	28-Feb-24	PX021148
Seq164	B50351	Nasal swab	Homo sapiens	Thailand	28-Feb-24	PX021149
Seq165	B50377	Nasal swab	Homo sapiens	Thailand	01-Mar-24	PX021150
Seq166	B50546	Nasal swab	Homo sapiens	Thailand	12-Mar-24	PX021151
Seq167	B52150	Nasal swab	Homo sapiens	Thailand	14-Jun-24	PX021152
Seq168	B55900	Nasal swab	Homo sapiens	Thailand	03-Dec-24	PX021153
Seq169	B55200	Nasal swab	Homo sapiens	Thailand	15-Oct-24	PX021154
Seq170	B54126	Nasal swab	Homo sapiens	Thailand	24-Aug-24	PX021155
Seq171	B50981	Nasal swab	Homo sapiens	Thailand	23-Mar-24	PX021156
Seq172	B50964	Nasal swab	Homo sapiens	Thailand	12-Apr-24	PX021157
Seq173	B50905	Nasal swab	Homo sapiens	Thailand	07-Apr-24	PX021158
Seq174	B50663	Nasal swab	Homo sapiens	Thailand	20-Mar-24	PX021159
Seq175	B50616	Nasal swab	Homo sapiens	Thailand	17-Mar-24	PX021160
Seq176	B50563	Nasal swab	Homo sapiens	Thailand	13-Mar-24	PX021161
Seq177	B50506	Nasal swab	Homo sapiens	Thailand	08-Mar-24	PX021162
Seq178	B50433	Nasal swab	Homo sapiens	Thailand	04-Mar-24	PX021163
Seq179	B50281	Nasal swab	Homo sapiens	Thailand	07-May-24	PX021164
Seq180	B50272	Nasal swab	Homo sapiens	Thailand	06-May-24	PX021165
Seq181	B50261	Nasal swab	Homo sapiens	Thailand	05-May-24	PX021166
Seq182	B50219	Nasal swab	Homo sapiens	Thailand	01-May-24	PX021167
Seq183	B50200	Nasal swab	Homo sapiens	Thailand	29-Apr-24	PX021168
Seq184	B50195	Nasal swab	Homo sapiens	Thailand	28-Apr-24	PX021169
Seq185	B50052	Nasal swab	Homo sapiens	Thailand	14-Apr-24	PX021170
Seq186	B50050	Nasal swab	Homo sapiens	Thailand	14-Apr-24	PX021171
Seq187	B49993	Nasal swab	Homo sapiens	Thailand	08-Apr-24	PX021172
Seq188	B49984	Nasal swab	Homo sapiens	Thailand	07-Apr-24	PX021173
Seq189	B49981	Nasal swab	Homo sapiens	Thailand	07-Apr-24	PX021174
Seq190	B49794	Nasal swab	Homo sapiens	Thailand	19-Mar-24	PX021175
Seq191	B49775	Nasal swab	Homo sapiens	Thailand	17-Mar-24	PX021176
Seq192	B49723	Nasal swab	Homo sapiens	Thailand	12-Mar-24	PX021177
Seq193	B49711	Nasal swab	Homo sapiens	Thailand	11-Mar-24	PX021178
Seq194	B49699	Nasal swab	Homo sapiens	Thailand	10-Mar-24	PX021179
Seq195	B49649	Nasal swab	Homo sapiens	Thailand	05-Mar-24	PX021180
Seq196	B49392	Nasal swab	Homo sapiens	Thailand	20-Feb-24	PX021181
Seq197	B48986	Nasal swab	Homo sapiens	Thailand	04-Feb-24	PX021182
Seq198	B48984	Nasal swab	Homo sapiens	Thailand	04-Feb-24	PX021183
Seq199	B48962	Nasal swab	Homo sapiens	Thailand	03-Feb-24	PX021184
Seq200	B48940	Nasal swab	Homo sapiens	Thailand	02-Feb-24	PX021185
Seq201	B48123	Nasal swab	Homo sapiens	Thailand	05-Jan-24	PX021186

Seq202	B48028	Nasal swab	Homo sapiens	Thailand	01-Jan-24	PX021187
Seq203	B48137	Nasal swab	Homo sapiens	Thailand	05-Jan-24	PX021188
Seq204	B48455	Nasal swab	Homo sapiens	Thailand	15-Jan-24	PX021189
Seq205	B48588	Nasal swab	Homo sapiens	Thailand	22-Jan-24	PX021190
Seq206	B48767	Nasal swab	Homo sapiens	Thailand	28-Jan-24	PX021191
Seq207	B51327	Nasal swab	Homo sapiens	Thailand	05-May-24	PX021192
Seq208	B55427	Nasal swab	Homo sapiens	Thailand	03-Nov-24	PX021193
Seq209	B49325	Nasal swab	Homo sapiens	Thailand	17-Feb-24	PX021194
Seq210	B50426	Nasal swab	Homo sapiens	Thailand	03-Mar-24	PX021195
Seq211	B52421	Nasal swab	Homo sapiens	Thailand	23-Jun-24	PX021196
Seq212	B55792	Nasal swab	Homo sapiens	Thailand	22-Nov-24	PX021197
Seq213	B50814	Nasal swab	Homo sapiens	Thailand	31-Mar-24	PX021198
Seq214	B50799	Nasal swab	Homo sapiens	Thailand	29-Mar-24	PX021199
Seq215	B50772	Nasal swab	Homo sapiens	Thailand	27-Mar-24	PX021200
Seq216	B50623	Nasal swab	Homo sapiens	Thailand	18-Mar-24	PX021201
Seq217	B50599	Nasal swab	Homo sapiens	Thailand	15-Mar-24	PX021202
Seq218	B50587	Nasal swab	Homo sapiens	Thailand	15-Mar-24	PX021203
Seq219	B50504	Nasal swab	Homo sapiens	Thailand	08-Mar-24	PX021204
Seq220	B50502	Nasal swab	Homo sapiens	Thailand	08-Mar-24	PX021205
Seq221	B50489	Nasal swab	Homo sapiens	Thailand	06-Mar-24	PX021206
Seq222	B50485	Nasal swab	Homo sapiens	Thailand	06-Mar-24	PX021207
Seq223	B50479	Nasal swab	Homo sapiens	Thailand	06-Mar-24	PX021208
Seq224	B50469	Nasal swab	Homo sapiens	Thailand	05-Mar-24	PX021209
Seq225	B50446	Nasal swab	Homo sapiens	Thailand	04-Mar-24	PX021210
Seq226	B50231	Nasal swab	Homo sapiens	Thailand	02-May-24	PX021211
Seq227	B50215	Nasal swab	Homo sapiens	Thailand	30-Apr-24	PX021212
Seq228	B49795	Nasal swab	Homo sapiens	Thailand	19-Mar-24	PX021213
Seq229	B49693	Nasal swab	Homo sapiens	Thailand	09-Mar-24	PX021214
Seq230	B49679	Nasal swab	Homo sapiens	Thailand	08-Mar-24	PX021215
Seq231	B49782	Nasal swab	Homo sapiens	Thailand	18-Mar-24	PX021216
Seq232	B50030	Nasal swab	Homo sapiens	Thailand	12-Apr-24	PX021217
Seq233	B50128	Nasal swab	Homo sapiens	Thailand	22-Apr-24	PX021218
Seq234	B50178	Nasal swab	Homo sapiens	Thailand	27-Apr-24	PX021219
Seq235	B50241	Nasal swab	Homo sapiens	Thailand	03-May-24	PX021220
Seq236	B50245	Nasal swab	Homo sapiens	Thailand	03-May-24	PX021221
Seq237	B50260	Nasal swab	Homo sapiens	Thailand	05-May-24	PX021222
Seq238	B55058	Nasal swab	Homo sapiens	Thailand	07-Oct-24	PX021223
Seq239	B54983	Nasal swab	Homo sapiens	Thailand	03-Oct-24	PX021224
Seq240	B51949	Nasal swab	Homo sapiens	Thailand	06-Jun-24	PX021225
Seq241	B48699	Nasal swab	Homo sapiens	Thailand	25-Jan-24	PX021226
Seq242	B48949	Nasal swab	Homo sapiens	Thailand	03-Feb-24	PX021227

Seq243	B54582	Nasal swab	Homo sapiens	Thailand	14-Sep-24	PX021228
Seq244	B54494	Nasal swab	Homo sapiens	Thailand	09-Sep-24	PX021229
Seq245	B53875	Nasal swab	Homo sapiens	Thailand	14-Aug-24	PX021230
Seq246	B53732	Nasal swab	Homo sapiens	Thailand	09-Aug-24	PX021231
Seq247	B53678	Nasal swab	Homo sapiens	Thailand	07-Aug-24	PX021232
Seq248	B53286	Nasal swab	Homo sapiens	Thailand	23-Jul-24	PX021233
Seq249	B53218	Nasal swab	Homo sapiens	Thailand	21-Jul-24	PX021234
Seq250	B53022	Nasal swab	Homo sapiens	Thailand	13-Jul-24	PX021235
Seq251	B52979	Nasal swab	Homo sapiens	Thailand	12-Jul-24	PX021236
Seq252	B52683	Nasal swab	Homo sapiens	Thailand	02-Jul-24	PX021237
Seq253	B52623	Nasal swab	Homo sapiens	Thailand	30-Jun-24	PX021238
Seq254	B52425	Nasal swab	Homo sapiens	Thailand	23-Jun-24	PX021239
Seq255	B51935	Nasal swab	Homo sapiens	Thailand	06-Jun-24	PX021240
Seq256	B51867	Nasal swab	Homo sapiens	Thailand	03-Jun-24	PX021241
Seq257	B51222	Nasal swab	Homo sapiens	Thailand	27-Apr-24	PX021242
Seq258	B51216	Nasal swab	Homo sapiens	Thailand	27-Apr-24	PX021243
Seq259	B49831	Nasal swab	Homo sapiens	Thailand	23-Mar-24	PX021244
Seq260	B49207	Nasal swab	Homo sapiens	Thailand	12-Feb-24	PX021245
Seq261	B48311	Nasal swab	Homo sapiens	Thailand	12-Jan-24	PX021246
Seq262	B48697	Nasal swab	Homo sapiens	Thailand	25-Jan-24	PX021247
Seq263	B48754	Nasal swab	Homo sapiens	Thailand	27-Jan-24	PX021248
Seq264	B48840	Nasal swab	Homo sapiens	Thailand	30-Jan-24	PX021249
Seq265	B49625	Nasal swab	Homo sapiens	Thailand	02-Mar-24	PX021250
Seq266	B51071	Nasal swab	Homo sapiens	Thailand	19-Apr-24	PX021251
Seq267	B51948	Nasal swab	Homo sapiens	Thailand	06-Jun-24	PX021252
Seq268	B52003	Nasal swab	Homo sapiens	Thailand	08-Jun-24	PX021253
Seq269	B52789	Nasal swab	Homo sapiens	Thailand	06-Jul-24	PX021254
Seq270	B52864	Nasal swab	Homo sapiens	Thailand	09-Jul-24	PX021255
Seq271	B52870	Nasal swab	Homo sapiens	Thailand	09-Jul-24	PX021256
Seq272	B52892	Nasal swab	Homo sapiens	Thailand	10-Jul-24	PX021257
Seq273	B53026	Nasal swab	Homo sapiens	Thailand	14-Jul-24	PX021258
Seq274	B54614	Nasal swab	Homo sapiens	Thailand	15-Sep-24	PX021259
Seq275	B54776	Nasal swab	Homo sapiens	Thailand	21-Sep-24	PX021260
Seq276	B51864	Nasal swab	Homo sapiens	Thailand	03-Jun-24	PX021261
Seq277	B53214	Nasal swab	Homo sapiens	Thailand	21-Jul-24	PX021262
Seq278	B53407	Nasal swab	Homo sapiens	Thailand	28-Jul-24	PX021263
Seq279	B54661	Nasal swab	Homo sapiens	Thailand	17-Sep-24	PX021264
Seq280	B54524	Nasal swab	Homo sapiens	Thailand	11-Sep-24	PX021265
Seq281	B54401	Nasal swab	Homo sapiens	Thailand	06-Sep-24	PX021266
Seq282	B53969	Nasal swab	Homo sapiens	Thailand	18-Aug-24	PX021267
Seq283	B53934	Nasal swab	Homo sapiens	Thailand	16-Aug-24	PX021268

Seq284	B53085	Nasal swab	Homo sapiens	Thailand	16-Jul-24	PX021269
Seq285	B52938	Nasal swab	Homo sapiens	Thailand	11-Jul-24	PX021270
Seq286	B52908	Nasal swab	Homo sapiens	Thailand	10-Jul-24	PX021271
Seq287	B52698	Nasal swab	Homo sapiens	Thailand	03-Jul-24	PX021272
Seq288	B52606	Nasal swab	Homo sapiens	Thailand	29-Jun-24	PX021273
Seq289	B52569	Nasal swab	Homo sapiens	Thailand	28-Jun-24	PX021274
Seq290	B52553	Nasal swab	Homo sapiens	Thailand	28-Jun-24	PX021275
Seq291	B52517	Nasal swab	Homo sapiens	Thailand	26-Jun-24	PX021276
Seq292	B52447	Nasal swab	Homo sapiens	Thailand	24-Jun-24	PX021277
Seq293	B52287	Nasal swab	Homo sapiens	Thailand	18-Jun-24	PX021278
Seq294	B51719	Nasal swab	Homo sapiens	Thailand	28-May-24	PX021279
Seq295	B51423	Nasal swab	Homo sapiens	Thailand	13-May-24	PX021280
Seq296	B51401	Nasal swab	Homo sapiens	Thailand	11-May-24	PX021281
Seq297	B51391	Nasal swab	Homo sapiens	Thailand	10-May-24	PX021282
Seq298	B51170	Nasal swab	Homo sapiens	Thailand	24-Apr-24	PX021283
Seq299	B51111	Nasal swab	Homo sapiens	Thailand	21-Apr-24	PX021284
Seq300	B51045	Nasal swab	Homo sapiens	Thailand	17-Apr-24	PX021285
Seq301	B51004	Nasal swab	Homo sapiens	Thailand	15-Apr-24	PX021286
Seq302	B50381	Nasal swab	Homo sapiens	Thailand	01-Mar-24	PX021287
Seq303	B49443	Nasal swab	Homo sapiens	Thailand	22-Feb-24	PX021288
Seq304	B49333	Nasal swab	Homo sapiens	Thailand	18-Feb-24	PX021289
Seq305	B48331	Nasal swab	Homo sapiens	Thailand	14-Jan-24	PX021290
Seq306	B48690	Nasal swab	Homo sapiens	Thailand	25-Jan-24	PX021291
Seq307	B48730	Nasal swab	Homo sapiens	Thailand	26-Jan-24	PX021292
Seq308	B48737	Nasal swab	Homo sapiens	Thailand	26-Jan-24	PX021293
Seq309	B48766	Nasal swab	Homo sapiens	Thailand	27-Jan-24	PX021294
Seq310	B49321	Nasal swab	Homo sapiens	Thailand	17-Feb-24	PX021295
Seq311	B49574	Nasal swab	Homo sapiens	Thailand	26-Feb-24	PX021296
Seq312	B49590	Nasal swab	Homo sapiens	Thailand	28-Feb-24	PX021297
Seq313	B49630	Nasal swab	Homo sapiens	Thailand	03-Mar-24	PX021298
Seq314	B49633	Nasal swab	Homo sapiens	Thailand	03-Mar-24	PX021299
Seq315	B51003	Nasal swab	Homo sapiens	Thailand	15-Apr-24	PX021300
Seq316	B51339	Nasal swab	Homo sapiens	Thailand	06-May-24	PX021301
Seq317	B51629	Nasal swab	Homo sapiens	Thailand	25-May-24	PX021302
Seq318	B51691	Nasal swab	Homo sapiens	Thailand	27-May-24	PX021303
Seq319	B51761	Nasal swab	Homo sapiens	Thailand	30-May-24	PX021304
Seq320	B51878	Nasal swab	Homo sapiens	Thailand	04-Jun-24	PX021305
Seq321	B51899	Nasal swab	Homo sapiens	Thailand	04-Jun-24	PX021306
Seq322	B52158	Nasal swab	Homo sapiens	Thailand	14-Jun-24	PX021307
Seq323	B52205	Nasal swab	Homo sapiens	Thailand	15-Jun-24	PX021308
Seq324	B52336	Nasal swab	Homo sapiens	Thailand	22-Jun-24	PX021309

Seq325	B52506	Nasal swab	Homo sapiens	Thailand	26-Jun-24	PX021310
Seq326	B52554	Nasal swab	Homo sapiens	Thailand	28-Jun-24	PX021311
Seq327	B52707	Nasal swab	Homo sapiens	Thailand	03-Jul-24	PX021312
Seq328	B52971	Nasal swab	Homo sapiens	Thailand	12-Jul-24	PX021313
Seq329	B53017	Nasal swab	Homo sapiens	Thailand	14-Jul-24	PX021314
Seq330	B53040	Nasal swab	Homo sapiens	Thailand	14-Jul-24	PX021315
Seq331	B53368	Nasal swab	Homo sapiens	Thailand	26-Jul-24	PX021316
Seq332	B53615	Nasal swab	Homo sapiens	Thailand	04-Aug-24	PX021317
Seq333	B53656	Nasal swab	Homo sapiens	Thailand	06-Aug-24	PX021318
Seq334	B53814	Nasal swab	Homo sapiens	Thailand	12-Aug-24	PX021319
Seq335	B53833	Nasal swab	Homo sapiens	Thailand	12-Aug-24	PX021320
Seq336	B54102	Nasal swab	Homo sapiens	Thailand	23-Aug-24	PX021321
Seq337	B54820	Nasal swab	Homo sapiens	Thailand	23-Sep-24	PX021322
Seq338	B49610	Nasal swab	Homo sapiens	Thailand	01-Mar-24	PX021323
Seq339	B55078	Nasal swab	Homo sapiens	Thailand	08-Oct-24	PX021324
Seq340	B49609	Nasal swab	Homo sapiens	Thailand	01-Mar-24	PX021325
Seq341	B49492	Nasal swab	Homo sapiens	Thailand	24-Feb-24	PX021326
Seq342	B48768	Nasal swab	Homo sapiens	Thailand	28-Jan-24	PX021327
Seq343	B48953	Nasal swab	Homo sapiens	Thailand	03-Feb-24	PX021328
Seq344	B49317	Nasal swab	Homo sapiens	Thailand	17-Feb-24	PX021329
Seq345	B52196	Nasal swab	Homo sapiens	Thailand	15-Jun-24	PX021330
